# Information Theory in Neuroscience

**DOI:** 10.3390/e21010062

**Published:** 2019-01-14

**Authors:** Eugenio Piasini, Stefano Panzeri

**Affiliations:** 1 Computational Neuroscience Initiative and Department of Physics and Astronomy, University of Pennsylvania, Philadelphia, PA 19104, USA; 2 Neural Computation Laboratory, Center for Neuroscience and Cognitive Systems @UniTn, Istituto Italiano di Tecnologia, 38068 Rovereto (TN), Italy

**Keywords:** information theory, neuroscience

## Abstract

This is the Editorial article summarizing the scope and contents of the Special Issue, Information Theory in Neuroscience.

As the ultimate information processing device, the brain naturally lends itself to be studied with information theory. Because of this, information theory [1] has been applied to the study of the brain systematically for many decades and has been instrumental in many advances. It has spurred the development of principled theories of brain function [2,3,4,5,6,7,8]. It has led to advances in the study of consciousness [9]. It has also led to the development of many influential neural recording analysis techniques to crack the neural code, that is to unveil the language used by neurons to encode and process information [10,11,12,13,14,15].

The influence of information theory on the study of neural information processing continues today in many ways. In particular, concepts from information theory are beginning to be applied to the large-scale recordings of neural activity that can be obtained with techniques such as two-photon calcium imaging to understand the nature of the neural population code [16]. Advances in experimental techniques enabling precise recording and manipulation of neural activity on a large scale now enable for the first time the precise formulation and the quantitative test of hypotheses about how the brain encodes and transmits across areas the information used for specific functions, and information theory is a formalism that plays a useful role in the analysis and design of such experiments [17].

This Special Issue presents twelve original contributions on novel approaches in neuroscience using information theory, and on the development of new information theoretic results inspired by problems in neuroscience. The original contributions presented in this Special Issue span a wide range of topics.

Two papers use the concept of maximum entropy [18] to develop maximum entropy models to measure the existence of functional interactions between neurons and understand their potential role in neural information processing [19,20]. Kitazono et al. [21] and Bonmati et al. [22] develop concepts relating information theory to measures of complexity and integrated information. These techniques have potential for a wide range of applications, not least of which is the study of how consciousness emerges from the dynamics of the brain. Other work uses information theory as a tool to investigate different aspects of brain dynamics, from *latching* in neural networks [23], to the long-term development dynamics of the human brain studied using functional imaging data [24], to rapid information processing possibly mediated by the synfire chains [25] that have been reported in studies of simultaneously-recorded spike trains [26]. Other studies attempt to bridge between information theory and the theory of inference [27] and of categorical perception mediated by representation similarity in neural activity [28]. One paper [29] uses the recently-developed framework of partial information decomposition [30] to investigate the origins of synergy and redundancy in information representations, a topic of strong interest for the understanding of how neurons in the brain work together to represent information [31]. Finally, the two contributions of Samengo and colleagues examine applications of information theory to two specific problems of empirical importance in neuroscience: how to define how relevant specific response features are in a neural code [32], and what the code used by neurons in the temporal lobe to encode information is [33].
